# Ethical Issues in Clinical Decision-Making about Involuntary Psychiatric Treatment: A Scoping Review

**DOI:** 10.3390/healthcare12040445

**Published:** 2024-02-09

**Authors:** Cláudio Domingos Laureano, Carlos Laranjeira, Ana Querido, Maria Anjos Dixe, Francisca Rego

**Affiliations:** 1 Psychiatric and Mental Health Service, Local Health Unit of the Leiria Region—Hospital of Santo André, Rua das Olhalvas, 2410-197 Leiria, Portugal; 2 Faculty of Medicine, University of Porto, 4200-319 Porto, Portugal; mfrego@med.up.pt; 3 Centre for Innovative Care and Health Technology (ciTechCare), Rua de Santo André-66-68, Campus 5, 13 Polytechnic University of Leiria, 2410-541 Leiria, Portugal; ana.querido@ipleiria.pt (A.Q.); maria.dixe@ipleiria.pt (M.A.D.); 4 School of Health Sciences, Polytechnic University of Leiria, Campus 2—Morro do Lena, Alto do Vieiro—Apart. 4137, 2411-901 Leiria, Portugal; 5 Comprehensive Health Research Centre (CHRC), University of Évora, 7000-801 Évora, Portugal; 6 Center for Health Technology and Services Research (CINTESIS), NursID, University of Porto, 4200-450 Porto, Portugal

**Keywords:** ethics, moral, involuntary psychiatric treatment, coercive measures, review

## Abstract

In mental health and psychiatric care, the use of involuntary psychiatric treatment for people with mental disorders is still a central and contentious issue. The main objective of this scoping review was to map and systematize the literature on ethical issues in clinical decision-making about involuntary psychiatric treatment. Five databases (Embase, PsycINFO, CINAHL, Medline, and Scopus) were searched for articles on this topic. Out of a total of 342 articles found, 35 studies from 14 countries were included based on the selection criteria. The articles were analyzed using the inductive content analysis approach. The following main categories were identified: (1) ethical foundations that guide clinical decision-making; (2) criteria for involuntary psychiatric treatment; (3) gaps, barriers, and risks associated with involuntary psychiatric treatment; (4) strategies used to reduce, replace, and improve the negative impact of involuntary treatment; and (5) evidence-based recommendations. Most of the selected articles discuss the logic underlying involuntary treatment of the mentally ill, exploring ethical principles such as autonomy, beneficence, non-maleficence, or justice, as well as how these should be properly balanced. During the process of involuntary psychiatric admission, there was a notable absence of effective communication and a significant power imbalance that disenfranchised those seeking services. This disparity was further intensified by professionals who often use coercive measures without a clear decision-making rationale and by family members who strongly depend on hospital admission. Due to the pluralistic and polarized nature of opinions regarding legal capacity and the complexity and nuance of involuntary admission, further studies should be context-specific and based on co-production and participatory research.

## 1. Introduction

Involuntary psychiatric treatment is one of the most controversial topics in contemporary psychiatry, a legacy of its institutional history but whose benefits remain difficult to assess. Although most mentally ill individuals do not experience coercive care, involuntary treatment is a universal experience in mental health services [1,2], deserving growing interest in health ethics. Involuntary treatment is simultaneously a common practice and almost the only exception to the principle that healthcare is voluntary and based on consent [3]. The use of coercive measures threatens the patient’s autonomy. Although they are usually used to help the patient, they can also be used to protect other people or even used abusively by healthcare professionals, thus becoming a morally complicated undertaking [4].

According to the Involuntary Admission and Treatment checklist devised by the World Health Organization (WHO) [5], most countries have similar conditions for detaining individuals. These conditions include the presence of a severe mental disorder and the requirement that compulsory treatment only be used to ensure the patient’s well-being or safety, or to protect others. Although several countries worldwide are striving to improve psychiatric practices and legislation, there is significant diversity. In all nations, a fundamental prerequisite is that the patient must have a mental condition. However, the specific type and degree of mental disorder that makes an individual eligible for involuntary admission differ among different legal jurisdictions. Certain nations permit forced entry exclusively in cases of “severe mental disorder (illness)”; in contrast, some countries require particular mental illnesses such as “psychotic illness” for involuntary hospitalization. The remaining countries employ a more expansive definition of mental disorder [6].

A recent meta-analysis, which included 77 studies conducted in 22 countries throughout the globe, revealed that 23% of the patients were admitted to the hospital against their will. It also indicated that involuntary psychiatric hospitalization is significantly associated with psychotic disorder, prior involuntary hospitalization, and male individuals who are unmarried, unemployed, receive welfare benefits, and lack residence [7]. People with severe and persistent mental illnesses (like schizophrenia or other psychoses) constitute the largest group admitted involuntarily; they account for 30–50% of all involuntary placements in countries that provide diagnostic information. Other involuntarily admitted groups include people with secondary diseases such as dementia, mood disorders, or drug abuse [8]. Furthermore, Sheridan Rains et al. [9] assert that nations with higher income levels and more availability of mental inpatient services had higher rates of involuntary hospitalization.

For more than three centuries, various ethical aspects of the methods used in psychiatric practice have been debated. Until the mid-twentieth century, mental health legislation reflected a paternalistic approach to involuntary psychiatric treatment. However, the last few decades have witnessed a development towards greater patient self-determination [10]. The serious effects on autonomy and individual freedom make coercion an intrinsically moral phenomenon [11], encompassing major dilemmas (such as forced medication or the use of restraints) and more everyday moral issues. Complex ethical challenges persist in this area, particularly when determining whether an individual is capable of making decisions independently, attesting to the moral complexity in the work of mental health professionals. 

The available data suggest that interventions focused on person-centered care planning and increased involvement of patients in decision-making [12] may have significant long-term effects on individuals undergoing involuntary psychiatric treatment, such as readmission rates [13,14]. The implementation of person-centered care (PCC) has been proposed as a strategy to effectively address the needs of individuals with complex mental health issues while also improving the efficiency, quality, and ethical standards of treatment [15,16]. The concept of value-based care lacks a precise and universally agreed-upon definition. Various interpretations of PCC have been proposed, emphasizing its holistic nature and its focus on recognizing the individual as a unique entity. These interpretations also highlight PCC’s aim to address the challenges faced in daily life, recognizing the person as an authority on their own experiences and acknowledging the person’s identity beyond their illness [17]. Sharing healthcare decisions is difficult due to structural power imbalances between patients and caregivers [18,19]. However, there are significant added difficulties when it comes to forced inpatient treatment since many of the patients’ options have been restricted. Involuntary inpatient facilities, voluntary inpatient settings, and outpatient clinics face distinct challenges when implementing shared decision-making [6,20,21]. If a physician determines that an individual cannot provide informed consent, a substitute decision-maker must choose treatment on behalf of the patient. Typically, this substitute decision-maker is either a close family member or a patient’s friend. Ideally, this person should be chosen by the patient. However, if the patient does not have a strong social network, a legal guardian or the court can fulfill this position [22].

Western psychiatry has been moving away from institutionalizing people with mental illness since the 1950s [23]. As the asylum movement crumbled, community-based treatment facilities gradually came to dominate psychiatric practice. With an increased focus on patient autonomy in medical decision-making and the introduction of Compulsory Community Treatment (CCT; also referred to as mandatory outpatient treatment or assisted outpatient treatment) for psychiatric practice worldwide (such as in the USA, Canada, Australia, New Zealand, Asia, UK, and the Netherlands), there has been renewed interest in this area, but it remains a highly contentious issue [24,25]. The main purpose of court-ordered therapy is to provide a less onerous alternative to involuntary admission, and to avoid relapses and readmissions (when there are issues like treatment non-compliance). Patients must adhere to certain requirements, such as taking prescribed medication and attending appointments, even if they stay in the community. Non-compliance with these stipulations typically leads to rehospitalization in a psychiatric facility [26,27], especially among those commonly referred to as “revolving-door patients” who struggle to maintain their recovery and adhere to prescribed treatment regimens, thereby requiring recurrent hospitalization [28,29]. Coercive outpatient programs are considered less restrictive than hospitalization, aiming to enhance individual autonomy. Nevertheless, the use of such programs has elicited varying perspectives within the mental health domain. Critics of CCT express apprehension over potential encroachments upon patients’ personal freedoms and potential harm to the therapeutic partnership [30]. Another area of concern is the possible transformation of CCT from a mechanism that promotes individual freedom to a treatment regimen that exerts control [28]. Nevertheless, the dearth of empirical support for CCT’s effectiveness in decreasing readmission rates or enhancing overall quality of life constitutes a significant counterargument.

While the United Nations Convention on the Rights of Persons with Disabilities (CRPD) drafted an important treaty defending the rights of persons with disabilities (including mental illness) [31], incorporating a human rights-based model of mental illness continues to face enormous challenges. Few studies have explored the possible role of ethics in programs to reduce coercive practices. However, determining when involuntary treatment can be effective—ensuring more consensual mental healthcare that does not compromise human rights—remains an enormous challenge [32,33,34]. Despite research examining the ethics of involuntary psychiatric treatment from various perspectives in recent years, there is still a lack of comprehensive scoping reviews that include this field. 

Based on these assumptions, the main objective of this scoping review is to map and systematize the literature on ethical issues in clinical decision-making about involuntary psychiatric treatment. The secondary objectives were: (a) to map the ethical foundations used in the literature to justify or reject the use of involuntary psychiatric treatment; (b) to examine the associated risks and address the ways and measures used to reduce, replace, and improve the negative impact of mandatory treatment in different clinical settings; and (c) to summarize and analyze the research results and highlight gaps in the evidence base. Our findings seek to provide a comprehensive synthesis of the current literature and offer a detailed informative basis for ethical ways to use involuntary psychiatric treatment. 

## 2. Materials and Methods

A scoping review provides the groundwork for further investigation, eventually contributing knowledge for policymaking and service provision. Thus, this scoping review was structured according to Levac et al.’s [35] enhancement to Arksey and O’Malley’s [36] five-step methodological framework for conducting scoping reviews. The phases of the framework are: (1) defining questions, (2) identifying relevant literature, (3) selecting studies, (4) extracting data, and (5) collecting, synthesizing, and reporting results. 

This review is reported following the Systematic Review and Meta-Analysis (PRISMA) Reporting Guidelines Extended to Scoping Reviews (PRISMA–ScR) [37]. Moreover, the review was registered in the Open Science Framework (OSF) (registration at https://doi.org/10.17605/OSF.IO/GZWVP accessed on 1 December 2023) following the JBI (Joanna Briggs Institute, The University of Adelaide, Adelaide, Australia) guidelines.

### 2.1. Definition of Research Question 

Based on recommendations by Colquhoun et al. [38], the research questions for this review were developed collaboratively by our research team consisting of one psychiatrist (C.D.L.) and four faculty members (C.L., A.Q., M.A.D., and F.R.). The notion of involuntary psychiatry treatment, as presented by Huber and Schneeberger [2], is a clinical strategy used to protect mentally ill persons, health personnel, and the general public from preventable aggression and violence. Thus, the main question of the review was: What are the ethical foundations described in the literature that guide clinical decision-making about involuntary psychiatric treatment?

The sub-questions were:

- What criteria are applied to justify or reject involuntary psychiatric treatment?
- What are the gaps, barriers, and risks associated with involuntary psychiatric treatment?
- What strategies are used to reduce, replace, and improve the negative impact of involuntary psychiatric treatment?
- What are the evidence-based or best practice recommendations for using involuntary psychiatric treatment?

### 2.2. Identification of Relevant Literature

Search terms were combined and refined using Boolean logic and proximity operators. All search terms used in this study were derived from Medical Subject Headings (MeSH) and CINHAL Headings. The search strategy was initially formulated for Medline searches (see Table 1), then adapted for Embase, PsycINFO, CINAHL, Medline, and Scopus. These databases cover the breadth of disciplines in this field. The grey literature was searched, focusing on official information channels such as international and government sources and professional bodies representing public healthcare and mental health. In addition, the reference lists of the included studies were inspected manually for additional references. The initial search was performed in May 2023 and updated in September 2023.

### 2.3. Study Selection Process

Peer-reviewed articles were screened from January 2008 through August 2023. The review timeframe was set from 2008 onwards, that is, since the United Nations Convention on the Rights of Persons with Disabilities [31] was entered into force. The Convention is “a human rights instrument with an explicit social development dimension. It adopts a broad categorization of persons with disabilities and reaffirms that all persons with all types of disabilities (including those with mental health disorders) must enjoy all human rights and fundamental freedoms” [31] (p. 1).

Sources were included when they met the eligibility criteria: (1) they examined ethical issues about involuntary psychiatric treatment in different clinical settings from any country; (2) they comprised empirical studies (qualitative, quantitative, as well as mixed studies), literature reviews, teaching articles, and conceptual/theoretical papers; (3) they were in English, Portuguese, and Spanish (the languages spoken by the research team). Furthermore, no geographical limitations were applied to the search strategy. Books and book chapters, dissertations, and conference proceedings were excluded.

Inclusion criteria were applied to articles retrieved via predetermined data-driven searches. These documents were imported into a bibliography management system. Duplicates were eliminated using Mendeley’s automatic duplication removal function. Articles that evaded software detection were eliminated manually following additional scrutiny.

During the review process, two reviewers conducted a two-stage assessment of each paper independently, according to the qualifying criteria. The first phase included the evaluation of the title and abstract, while the subsequent phase entailed a comprehensive analysis of the chosen articles. Any article considered relevant by at least one reviewer advanced to the later stages of the review process. Then, two blind readers conducted an evaluation and assessment of the full-text articles. Only publications deemed relevant by both readers were included in the present study.

To maintain consistency and precision in the selection process, the inter-rater reliability of both titles/abstracts and full-text articles was assessed using the percentage of agreement. When agreement achieved a threshold of more than 80% among the team members, the subsequent research stage was initiated. All instances of disagreement between the two reviewers were thoroughly deliberated and addressed via discussion, leading to a consensus. When unanimity was deemed necessary, a senior reviewer was consulted to provide a third viewpoint.

### 2.4. Data Extraction and Analysis

The main themes derived from the chosen articles were classified, succinctly outlined, and visually represented in the form of data charts. The information was graphed, and essential details were input into a table under the following headings: title, authors, and year; region; study design; purpose; and significant findings. Maintaining flexibility, the charting form was revised as required during the charting procedure.

The findings were collated, synthesized, and reported to produce a thorough review and summary of the body of research on ethical issues in involuntary psychiatric treatment. Key conclusions were summarized using a narrative synthesis. This scoping review used descriptive qualitative content analysis to present the data extracted from the included studies. To be more precise, the basic features and conclusions of the included research (aggregative synthesis) were discussed before presenting a summary of the conclusions from all the included investigations (configurative synthesis) [36]. The main findings are organized based on this scoping review’s questions and were categorized and reported in five relevant categories: (i) ethical foundations that guide clinical decision-making; (ii) criteria for involuntary psychiatric treatment; (iii) gaps, barriers, and risks associated with involuntary psychiatric treatment; (iv) strategies used to reduce, replace, and improve the negative impact of involuntary treatment; and, (v) evidence-based recommendations for the use of involuntary psychiatric treatment. The retrieved data are also shown in a tabular and diagrammatic manner. A narrative overview is provided alongside the tabulated findings.

According to the standards for scoping reviews, no meta-analysis, quality assessment, or risk of bias assessment was performed for this scoping study [39].

## 3. Results

**Figure 1:**
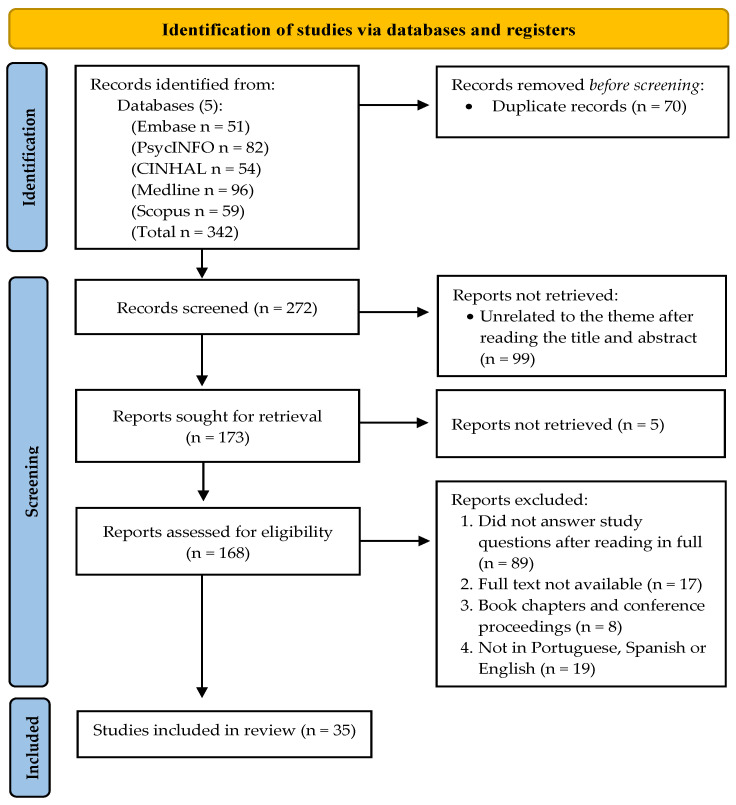
Study flow diagram with search strategy using PRISMA guidelines.

Four review phases were carried out per the PRISMA–ScR flow diagram: identification, screening, eligibility assessment, and final synthesis (see Figure 1). 

Our search string initially yielded a total of 342 articles. The removal of duplicates and the initial screening resulted in 272 articles. Ninety-nine articles were removed after a preliminary screening of their titles and abstracts. The remaining 173 full-text articles were then reviewed (five reports were unretrievable). After applying our exclusion criteria, a final total of 35 papers were included. The extraction results for the study’s specific features are listed in Table 2.

Most of the papers were original research (n = 20; 57.1%), ten were discussion papers, and five were review articles. The original evidence mainly used a qualitative paradigm (n = 15). Papers focused on a particular country, including Norway (10), the United Kingdom (4), Sweden (4), New Zealand (3), Switzerland (2), USA (2), the Netherlands (2), and Canada (2), and one from Australia, Ireland, Slovenia, Belgium, South of Africa, and South Korea (Table 2).

### 3.1. Ethical Foundations That Guide Clinical Decision-Making

Most of the selected articles discuss the logic underlying the involuntary treatment of the mentally ill, exploring ethical principles such as autonomy, beneficence, non-maleficence, or justice [40,41,42,43,44,45], as well as how these should be properly balanced. Several authors defend that an individual who suffers from a mental illness but can decide whether to undergo treatment should be allowed to choose, except when serious harm to health is likely to occur without this treatment [40]. Suppose the mental illness seriously impairs a patient’s decision-making capacity and the treatment is considered necessary; in that case, it is legitimate to assume that treatment is in the patient’s interest, allowing them to promote or recover their autonomy and human dignity [40,41].

Some authors recognize that depriving capable people of their right to make decisions is a form of abuse, but failing to recognize a lack of capacity may result in continued vulnerability [46]. Some authors argue that coercion should only be used for the benefit of the patient and not exclusively to prevent danger to others, which is always difficult to predict [47]. It is equally arguable that, since the risk to third parties is so evident in certain circumstances, priority should be given to the principles of non-maleficence and justice [43,48]. Coercive actions to prevent risks to third parties are not the same as punishing a person for some infringement, a role that is completely unrelated to health professionals [44]. In contrast, one study considers it unethical to abandon a non-dangerous mental patient who needs care and may benefit from support but does not meet the conditions for involuntary treatment [49].

Another perspective argues that for involuntary treatment “to be ethically justified and supersede a patient’s autonomy, one must be able to demonstrate that they are incapable of knowing what is in his/her own interest and that the therapeutic team is capable of such assessment” [42] (p. 130), based on the literature and an understanding of the patient’s desires and preferences [42,50]. From an ethical standpoint, a lack of scientific evidence regarding the effectiveness of a particular treatment does not automatically render it unethical [44].

Regarding involuntary treatment, some articles argue that health professionals should resort to certain ethical guidelines, namely dominant rights and ethics of care, such as relational ethics [44,48,51,52]. The rights-based ethical approach assumes people are autonomous, where everyone’s personality and humanity have exactly the same intrinsic value, and it also defines acts that infringe upon a person’s autonomy and the criteria for considering a patient incompetent and/or dangerous [52]. The relational ethical approach, on the other hand, promotes the maintenance of an ethical relationship within the web of influences inherent to the clinical relationship, defends congruence with the lived experience of clinical situations, and recognizes health professionals (with their moral intuition) and patients as distinct and specific persons [52]. New concepts, such as relational autonomy, can also help see coercion as a care practice that aims to protect the patient, despite the temporary annulment of their self-determination, if the aim is the long-term recovery of autonomy [44]. However, given the current limited resources, adopting a relational ethics approach can be a difficult challenge in the context of psychiatric practice [51], namely in promoting a balance between respect for an individual’s autonomy and the health professional’s moral obligation to decide when a patient is unable to decide competently in their best interest [51].

Interpretive dialogue and the promotion of dignity are also considered central aspects, as the method of assessment influences patients’ perception of coercion and, subsequently, their motivation to fully participate in treatment [48]. This debate also involves fundamental philosophical arguments between deontological and utilitarian ethics concerning whether it is ethically justifiable to restrict freedom and personal rights based on ‘benevolent’ coercion aimed at protecting society and promoting the individual’s right to treatment, a difficult position to reconcile with the current emphasis on personal autonomy [53]. An additional application of Mill’s classic utilitarian argument is to support the claim that coercion can be justified so long as it safeguards the patient’s fundamental rights to life and health. Nevertheless, this justification must be predicated on the patient’s best interests and implemented with minimal encroachment upon their autonomy [54].

Several selected studies assess various ethical challenges that mental health professionals face in the context of involuntary treatment [43,45,55,56,57,58,59,60,61,62]. Central factors explaining the attitudes expressed by professionals include a feeling of impotence, the desire to care for the patient, or the fear that abstaining from coercive interventions can lead to a more disastrous outcome [45]. A Norwegian study addresses the concept of ‘moral doubt’ in the context of involuntary treatment, admitting that not knowing or not being sure if something is morally correct or justified is not always positively valued, and can be seen as a sign of weakness or inexperience and even have a crippling effect by impeding or delaying decisions [59]. An Australian study concluded that participating professionals seemed to use the moral framework to justify the imposition of care, placing themselves in the patient’s shoes and acting virtuously with them, thus mitigating coercion and paternalism [57]. Certain authors acknowledge the occasional challenge of precisely distinguishing between professional and moral attributes due to their interdependence, contending that ethical and professional dilemmas are inextricably linked [55,56]. Other articles show that coercion involves individual and institutional ethical aspects, assuming that it often induces psychosocial tension in the therapeutic team. Various types of systematic reflection, moral deliberation, and ethical support can help face emerging challenges, improving cooperation between multidisciplinary team members by providing more systematic ways of understanding and dealing with moral issues [43,60,62].

Regarding the relevant ethical values for patients undergoing involuntary treatment, some articles highlight issues such as freedom of choice, autonomy, participation in the decision-making process, and a sense of security during hospitalization, in addition to the need to receive sufficient information to be heard and treated with respect, trust, and in a non-paternalistic way [55,56,63,64]. Other factors identified as influential for patients include their perception of the team’s attitude and whether they have the necessary qualifications to provide good care (beneficence), and the expectation of positive results, namely therapeutic value or contribution to the recovery process [55,56,63]. These elements underscore the significance of incorporating the ethical principles of proportionality and purpose into assessments of coercion. It is required that the extent of coercive intervention be limited to what is essential for the given circumstance, ensuring sufficient equilibrium. Furthermore, coercion should not be employed without a premeditated, unambiguously defined objective that is customized to the patient’s requirements and preferences and substantiated by evidence [63].

Regarding the existential and moral dilemmas of family members, one study suggests that coercion can reduce but also increase the family’s burden, increasing tensions in family relationships, dilemmas, (moral) anguish, and retrospective regrets. Lack of information or involvement may intensify these reactions, highlighting the potential influence of the mental health system [65]. Another article highlights that although other stakeholders, such as family members or neighbors, can initiate the admission process by alerting the authorities or health services, they are not considered part of the admission process, lacking any decision-making power [66].

Several articles focus on the ethical challenges inherent in involuntary community treatment [42,51,54,57,67,68,69]. This treatment modality’s central ethical issue, at least from a principled perspective, is the restriction on the patient’s autonomy [42]. A common justification is that the restrictions on the patient’s autonomy are smaller under this measure than they would be under hospitalization. However, the ethical risks imposed by restricting autonomy are more problematic in the community environment. When the patient is well enough to live in the community, the need to restrict autonomy will be more difficult to defend, making ethical issues opaque [42]. In fact, an illusion of greater freedom is created, and refusing to follow treatment may result in involuntary hospitalization, thus coercing the patient [42,67,68].

Finally, the decision to act based on a prior self-binding directive is also the subject of debate. Responsible practice requires assessing the patient’s decision-making capacity when creating the directive and subsequently when making a decision [70]. Another article argues that advanced psychiatric directives cannot prevent involuntary commitment when appropriate because they overlap with the duties of state police [64].

### 3.2. Criteria for Involuntary Psychiatric Treatment

Defining criteria for involuntary treatment is crucial to prevent abuse, as the two most common grounds for justifying this measure are the likelihood of imminent danger and the need for urgent treatment in a hospital [66]. Certain authors contend that the only justification for involuntary treatment is when it is in the patient’s best interest; that is, when a mentally ill individual poses a significant risk of physical or mental damage if left untreated, or when they pose a threat to themselves [40,63]. Dangerousness as a criterion to justify involuntary hospitalization has attracted some attention. Following the principle of the minimum use of force [47], some authors advocate that patients may be hospitalized involuntarily if they represent a threat to themselves or others [49,56,58,68,71], and only when there is a clear and objective prediction of potential damage. In these cases, it is generally argued that the potential danger will result from the mental illness, based on substantial evidence that the coerced individual will probably cause harm to someone or themselves; involuntary treatment [71] is considered the means to remove the danger [49]. Considering that many violent acts are committed by people without mental illness, other authors reject that involuntary treatment is justified to protect others, questioning why dangerousness should be a reason for preventive hospitalization of mentally ill patients [40,66]. Conversely, certain scholars argue that involuntary treatment does not inherently require a lack of capacity, given that the concept of decision-making capacity is not precisely defined in several health legislations [58]. Arguably, individuals with mental illness should receive treatment if they are deemed incompetent since it is in their best interest to prevent harm to others. However, even in such situations, regardless of potential dangers, treatment for the individual’s illness is still necessary [40]. This highlights the need for a comprehensive approach, including “the need for treatment criteria covering the more-frequent psychiatric clinical conditions, and danger criteria that could be used during less-frequent but severe and dangerous behavioral manifestations, which also need to be addressed by both law enforcement and clinical intervention” [75] (p. 5).

An additional group of reasons for consenting to involuntary treatment was related to promoting the patient’s autonomy by carrying out actions according to their authentic volition [58]. Nevertheless, certain authors argue against the use of coercive treatment for the mentally ill if the sole motivation is the patient’s health. While positive outcomes for the patient’s health may be a prerequisite for coercive treatment in psychiatry, they are not sufficient justification with mentally capable patients; thus, there is no justification for differentiating between psychiatric and physically ill patients [40]. However, while regard for human dignity and autonomy should not be the sole basis for rejecting coercive measures, the absence of controlled trials examining the positive outcomes of such measures undermines the case for involuntary treatment [41,51]. While autonomy was used as a reason to justify involuntary treatment, respect for self-determination was specifically invoked if the immediate risk of harm was considered low [58]. We should govern our actions under the precautionary principle of “primum non nocere” so long as there is no evidence of coercion’s positive effects [41]. An additional justification for refusing involuntary treatment was the potential erosion of the patient–physician alliance and the patient’s loss of confidence in psychiatric services as a result of the procedure’s restriction of autonomy or physical coercion [58,61]. Under the Stone and rehabilitation models, the moral basis for involuntary treatment is the revival of an individual’s autonomy, and three classes of mentally ill patients are treated involuntarily: dangerous patients whose clinical condition is treatable; dangerous patients within untreatable conditions; and finally, non-dangerous patients with treatable conditions [49].

Cases involving psychotic or suicidal patients, who are unaware of their care requirements, were generally regarded as prime illustrations of justified involuntary care [50,58,61]. Nevertheless, suicide was met with ambivalence, as some contended that the potential for suicide in itself might not be adequate justification for the proposed measure. Typically, “severe mental illness” pertains to euphoric states accompanied by psychotic symptoms or illness. Most laws, however, do not specify which pathologies are admitted. Consequently, personality disorders and other severe psychiatric pathologies (such as depression) may be classified as serious psychiatric disorders if the symptoms are sufficiently severe [58]. Various factors contribute to the exclusion or inclusion of personality disorders, and there is a lack of clinical consensus regarding impaired decision-making, risk assessment, and suitable treatment [48,72].

Regarding the criteria for CCT, it is important, from an ethical point of view, to bear in mind that any extension of the state’s power to act in the best interests of citizens depends on actions providing sufficient reciprocal benefits [67], but also that the measures impose as few restrictions as possible [54,67]. Many studies have revealed benefits for patients, particularly by encouraging adherence to medical treatment, facilitating access to certain social supports, and reducing hospital admissions [53,69].

When interventions based on advance directives occur, it is widely accepted that a competent patient’s refusal must be respected, implying that prior consent can always be revoked or adapted if the patient has decision-making capacity and so desires [70].

### 3.3. Gaps, Barriers, and Risks Associated with Involuntary Psychiatric Treatment

In the selected articles, one of the gaps associated with involuntary psychiatric treatment refers to the characteristics of the professional who decides on coercive measures, and other elements that affect decision-making, namely the caregiver’s personal values; the culture of the institution in which they work; the legal framework; previous decisions; and the opinions of colleagues, family members, and superiors [41,50,61]. One article concludes that, among nurses, greater academic differentiation increases the possibility of evaluating the pertinence of medical prescriptions and, consequently, how coercive measures are implemented during involuntary treatment [52]. Other described gaps refer to the legislation on involuntary treatment, whose vagueness can allow distortions, and to the shortage of professionals and hospital vacancies, as well as the difficulties when interpreting psychiatric diagnoses [46,50,61].

From the users’ perspective, the most frequently mentioned gaps result from the experience of not being heard, lacking information, and not being involved in decision-making about treatment plans; that is, when their dignity and respect are compromised [43,48,53,56,64]. Family members also point out this gap, highlighting the lack of involvement despite their availability in patient care [65]. Users also indicated the physical environment’s shortcomings and a lack of daily activities in the hospital [41,53,56]; their perception that coercion did not help them recover [54]; or the side effects of medication [64]. The finding that involuntary community treatment does not reduce readmission rates or their duration, nor does it increase readmission time or adherence to treatment, are other aspects described in the analyzed articles [57].

Understanding whether mental illness affects a psychiatric patient’s ability to make autonomous choices regarding treatment is considered a key barrier [40]. The absence of services with adequate response capacity can also cause a family to resort to involuntary hospitalization as the only available solution, even when they prefer voluntary alternatives [65]. There is a consensus that coercion is an intervention of last resort in psychiatry, but fully defining what last resort means may be challenging [73]. On the other hand, coercion is sometimes considered the main barrier to developing a therapeutic relationship between the patient and health professionals. The experience of compulsory hospitalization, however, can be modified by the therapeutic rapport, particularly when the patient feels the decision was made in their best interest to promote their safety [68].

There is some evidence that restraint reduction programs can improve the quality and safety of care but do not reduce the use of coercion [74]. The lack of knowledge about how various types of support services for clinical ethics may influence the quality of care and how patients and family members can evaluate these services may be seen as another relevant barrier to be overcome [62].

A relevant risk results from apparent discrimination against patients who are Black, migrant, from ethnic minorities, and with low socio-educational levels being hospitalized involuntarily compared to other populations [57,61,66,68,69]. Allegedly, involuntary treatment might be used to gain social control over vulnerable individuals rather than developing the intensive care programs they so badly need [50,69]. Another aspect results from the observation that most community treatments were initiated in the hospital, suggesting that the main objective was to shorten the length of hospital stay rather than preventing it [68]. Another risk results from coercion possibly harming family relationships, as patients tend to feel abandoned, and the family may experience a complex and traumatic process [65]. Some authors also warn that involuntary treatment may lead to a loss of self-confidence and impaired therapeutic interactions, severely interrupting any voluntary search for help [47,57,64,71]. The use of advance directives in patients is also considered a risk as they can be misused, particularly when pressure is exerted [50,70].

### 3.4. Strategies Used to Reduce, Replace, and Improve the Negative Impact of Involuntary Treatment

Reducing the use of involuntary treatment will certainly require a cultural change, involving patient-related issues but also many complex factors associated with health professionals and institutions [41]. Strategies that should be explored include assertive community treatment, personalized and shared care plans, early intervention in crises, and triage models that mitigate worsening mental health problems and lead to intervention before a crisis begins [51,65,74]. When coercion is justified based on clinical assessments, adhering to the principle of least harm to minimize any restrictions on freedom of action is of utmost importance [54].

To facilitate moral deliberations and foster the development of strategies to enhance therapeutic alliances and reduce coercion in the field of psychiatry, social processes can assume a significant role by introducing novel ideas in dialogue with patients [71]. Overcoming the moral gray zone paradox between caring and controlling requires that the patient be treated as an active moral agent in their recovery process [57]. Several authors advocate the patient’s full involvement in all stages of treatment, adopting a flexible and responsive approach to meet their intentions whenever possible and helping them promote their dignity [43,48,53,62,64,66]. The practice of involuntary treatment can be improved in terms of personalization but also relational aspects of care, where communication and the active participation of families are essential [48,53,66]. Another guideline to promote good practices suggests that professionals should have more opportunities to exchange knowledge and experiences with each other [53,61,62]. By increasing the team’s awareness and capacity to articulate their motivations for action, professional competence and reflexivity can be enhanced through ethical deliberation, moral discernment, and ethical considerations regarding how to apply fundamental principles and standards to concrete moral dilemmas [48,60]. By promoting awareness regarding the appropriate application of coercion and fostering improved collaboration, ethical think tanks can aid in the development of better procedures and facilitate a more methodical approach to morally challenging circumstances [60,62]. Health professionals who more frequently experience moral doubt were more critical of using coercive measures [59]. Improving society’s knowledge and perception of involuntary treatment will be equally crucial, as this is the only way to achieve necessary organizational and cultural change [62,74].

### 3.5. Evidence-Based Recommendations for the Use of Involuntary Psychiatric Treatment

Enforcement approaches determined via a transparent and meticulously balanced review process are more inclined to be suitable, comprehensible, and embraced by patients and caregivers [44]. Assuming that coercion is morally permissible under certain conditions, one must ascertain whether there are thresholds beyond which coercion becomes disproportionate or situations in which it is unacceptable [44]. Prior knowledge of existing arguments and associated controversies allows one to create adequate distance and make the right decisions when difficult situations arise. Considering coercion in a broader context is critical to understanding what is important from the patient’s perspective, assessing any concerns the patient may have regarding the treatment plan (including respect for their dignity), and allowing them to experience maximum self-determination (if possible, throughout the process), thus promoting the therapeutic relationship [48,56,63,69]. A deeper understanding of patients’ viewpoints may facilitate the resolution of moral problems in clinical practice or foster discussions among relevant stakeholders regarding complex ethical issues [63], whereas denial of rights may devalue and/or disregard the biography and abilities of users [56]. Involving the patient in the decision-making process is a critical part of the rehabilitation process, potentially increasing their motivation for treatment [48,61]. By considering coercive measures not solely as a means of reducing risk and ensuring security but also as an integral component of a medium-term process to reconstruct identity and autonomy, coercion processes can be rendered more universally acceptable [44].

Systematic ethical reflection in health services is a young discipline with great potential in mental health care [45,62]. Participation in ethical reflection groups seems to help remove hierarchies among professionals. In these groups, they recognize they are not alone in experiencing an ethical challenge and feel the conclusion belongs to everyone [62]. Establishing a methodical ethical reflection process, which includes all stakeholders (including patients, families, and professionals) in examining health challenges, is critical [49,55,62,65]. A strategy to open a moral space in health units could involve a more explicit use of formal support for clinical ethics [60]. Moral case deliberation may enhance an organization’s overall ethical standing by encouraging “reflection on good care in general and seclusion in particular” [43] (p. 9). Ethics can significantly benefit quality assurance and development projects involving coercion by providing more methodical approaches to moral issues. Integral and process-oriented ethics are essential due to the interconnectedness of organizational environments, professional facets, and moral concerns [59,60]. The creation of guidelines for legal and medical professionals, prepared with contributions from patients and family organizations, will encourage the use of good practices in involuntary hospitalization and promote voluntary treatment [57,66]. In addition, institutions should continually promote ethical sensitivity, empathy, and knowledge of legislation among health professionals, and the educational curriculum should emphasize ethical behavior as an important personal value [47,52,53,57].

Further empirical research is necessary to explore the claim that better therapeutic results may be achieved by using compulsive measures; a claim often used to challenge ethical concerns [68]. Increased awareness will promote the development of a mental health system that works for the individuals it serves [69]. The purpose of research in this field is not to determine whether involuntary treatment is “right or wrong” but to investigate when it can be effectively implemented and used to strengthen the therapeutic alliance in clinical practice [68].

## 4. Discussion

The present study contributes to the discussion on the ethics of involuntary treatment in psychiatry, where it remains one of the most controversial issues. Mental health is one of the few areas of medicine where involuntary treatment is both legally sanctioned and regularly used. It continues to raise ethical debates worldwide and is sometimes associated with negative consequences for patients and professionals, including compromising the therapeutic alliance. The benefits of coercive measures thus remain questionable, as they potentially jeopardize several fundamental human rights (such as freedom of choice or movement, autonomy, and physical integrity) and may even be seen as therapeutic failures [76]. With the increased focus on patient autonomy in medical decision-making [68] and the growing number of countries regulating involuntary community care, the discussion around the application of the Convention on the Rights of Persons with Disabilities (CRPD) added renewed interest in these issues [31]. Resorting to involuntary measures, while having the obligation to guarantee good healthcare, is a complex moral task, involving both classic dilemmas (such as autonomy versus paternalism) and more everyday issues. Constraining a patient’s freedom and dignity is controversial, and therefore, it is difficult to determine whether a specific coercive measure in a particular context is morally acceptable and what the decisive determinant is. Moral arguments against the use of coercive measures include the violation of the patient’s autonomy, dignity, and integrity. However, many authors justify the use of coercive measures as long as they reduce suffering and/or risk for the patient and improve, or even restore, their autonomy in the long term. Reluctance to use coercion may end up denying a patient the help and care they really need [59]. If autonomy is considered an absolute criterion, it can cause more harm than good, leaving a patient alone to make their own decisions and taking responsibility away from other actors. From an ethical point of view, the big question is finding a thoughtful balance between the protection of autonomy, individual rights, the health and safety of the mentally ill, as well as the public good, ensuring that the restriction of personal freedom will only occur when ethically and medically justifiable. For John Stuart Mill, a central issue is determining when society can legitimately exercise power over the individual. He argued that self-protection and the need to prevent harm to others are the only legitimate reasons to interfere with another person’s freedom of action. In line with the principles of beneficence and non-maleficence developed by Beauchamp and Childress, several authors [44,77] suggest that the arguments for and against must be weighed, such that the use of coercive measures is only morally acceptable when the ‘benefits’ of protection or treatment outweigh the ‘negative effects’ on autonomy and the integrity of patients. Assessing the balance between promoting good (beneficence) and inflicting harm (maleficence) is, therefore, a primary ethical challenge. Given the importance of this balance, the most important justification for coercion is the promotion of the patient’s best interest, as advocated by Beauchamp and Childress [78].

Evidence has shown that the implementation of person-centered and self-directed treatment methods, together with the integration of shared decision-making and psychiatric advance directives, enables patients to exert more autonomy and influence over their healthcare [79]. The aforementioned advantages are seen in levels of compliance and self-management, as well as in medical and psychological health results, resulting in enhanced cost-efficiency, satisfaction with services, and quality of life [80]. Additionally, there is a decrease in the use of inpatient and emergency department services, and perhaps a reduction in involuntary treatment [81]. The advantages seem to be more pronounced when therapies are implemented in a complete, intense, and integrated manner within ordinary healthcare practices [82].

Mental health professionals must be at the forefront in defending human rights and helping overcome stigma and discrimination. In a fair and equitable society that respects the right to social inclusion of individuals with mental illness and promotes prevention and early intervention in mental health, reducing the use of involuntary treatment is an achievable goal in mental health. However, classifying coercion in general as “unethical” is reductionist, neglecting the need for ethical and practical guidance in urgent situations where competing values must be weighed [83]. In a relentless search for consensus, ethical reasoning about competing options will therefore be crucial for making unprejudiced decisions that comply with the regulatory framework.

Further studies should examine the impact of universal health coverage and universal human rights indices on rates of involuntary admission. Given policymakers’ focus on cost-saving measures, it is also crucial to incorporate economic outcomes in research examining the efficacy of alternatives to involuntary treatment. Furthermore, it is imperative to conduct further research on the impact of seasonal patterns [84] and environmental variables on involuntary admission, particularly in the context of the escalating consequences of climate change [85]. From here, it will be possible to develop timely intervention and preventative methods to address these stressful hospitalizations.

### Strengths and Limitations

This scoping review has several shortcomings. One of the major limitations of this research is the subjectivity of the reviewers. This is a typical flaw in qualitative analysis. Another shortcoming is the lack of linguistic diversity in the publications chosen by the research team, since the included publications were predominantly in English. The number of published items that were screened may have been further hampered by the exclusion of books, book chapters, and conference proceedings. In addition, a small number of databases were searched. As a result, some important publications not included in the aforementioned five databases may have been overlooked. However, one strength of this study is the inclusion of both original research, narrative reviews, and discussion papers without regard to geography.

Cultural or legal aspects were not discussed. Considering the heterogeneity of articles and the topic under analysis, this may have conditioned some conclusions. In addition, no qualitative analysis of the selected studies was carried out.

While this scoping assessment had wide geographical coverage, it neglected to account for variations in cultural and legal systems across countries. In this respect, the legal implementation of involuntary treatment of the mentally ill may differ between person-centered care systems and the biomedical model of care. Therefore, more investigation is required for a more advanced comprehension of involuntary treatment from an ethical standpoint and how its implementation varies across care paradigms. Lastly, further studies should be context-specific and based on co-production and participatory research, reflecting the individual perspectives of all stakeholders, particularly patients, families, and healthcare professionals.

## 5. Conclusions

This scoping review is a contribution to the ongoing discourse around the ethical considerations of involuntary treatment within the field of psychiatry. By conducting a systematic assessment of the existing scientific literature, this research endeavors to enhance comprehension of prior studies and provide a more robust foundation for engaging in discussions about this topic. To improve mental health care, a systematic analysis of the ethical challenges when dealing with involuntary measures is necessary. Therefore, a continuous discussion is urgent; one that involves intrinsic self-critical reflection by all involved agents and explores the ethical and legal controversies surrounding the deprivation of liberty and the involuntary treatment of patients. Rather than seeking to reach a single answer, ethics requires a posture of constant questioning, where multiple perspectives and ambiguities are embraced.

An absence of effective communication and an asymmetry of power among stakeholders hinder service users’ rights, preferences, and will. The situation is further compounded when practitioners justify coercion, presuming that individuals receiving services cannot comprehend information. Service users may use various support services to address these issues, which, when complemented by a range of community resources, can uphold the autonomy of the service users. People with severe mental illness often experience high rates of withdrawal, non-adherence to treatment, involuntary admission, and chronic disease progression [86]. Therefore, shared decision-making treatments, such as collaborative crisis plans and self-management interventions with relapse prevention elements, are successful in minimizing involuntary hospitalizations [87,88].

The use of coercive tactics in mental health is generally acknowledged as a last recourse when all other options fail to achieve the desired outcomes, giving rise to multiple ethical challenges. However, paradoxically, ethical discussions about the tensions between coercion and respect for the fundamental rights of patients are understudied in psychiatry when, in reality, limitations to freedom occur more frequently in this context. The lack of data on the clinical benefit of involuntary hospitalization has been identified as a serious limitation. Further research is necessary to foster knowledge about ethical reflection and practice. Future studies should also focus on the best way to prepare new health professionals for this function, aiming to better guarantee patient autonomy.

## Figures and Tables

**Table 1:** Search strategy used in Medline for identifying potentially pertinent articles.

Key Concepts	TITLE-ABS-KEY
	[“coercion” OR “compulsory” OR “seclusion” OR “restraint” OR “coercive measure” OR “involuntary admission” OR “involuntary commitment” OR “involuntary treatment” OR “coercive care” OR “coercive treatment” OR “compulsory care” OR “compulsory treatment” OR “compulsory admission” OR “compulsory commitment”]
AND	[“psychiatry” OR “mental health” OR “community mental health services” OR “mental health services”]
AND	[“ethics” OR “ethical analysis” OR “bioethics” OR “ethical issues” OR “bioethical issues” OR “moral” OR “legitimacy”]

**Table 2:** Pertinent data extraction of articles reviewed.

Title/Author/Year	Country	Purpose	Article Type/Study Design	Key Findings
Coercive treatment and autonomy in psychiatry. (Sjöstrand and Helgesson, 2008) [40]	Sweden	Discusses the rationale behind coercive treatment, exploring the meaning and realm of autonomous choice.	Discussion paper	Involuntary treatment is not justified for the sake of protecting others, but only if the patient cannot make an autonomous decision and the treatment is in the patient’s genuine interest. Patients unable to make decisions for themselves may have valid reasons, based on their strongly held beliefs, to refuse mandatory institutional care. These reasons should be honored unless it can be presumed that their current circumstances influenced a change in their perspective.
Can we justify the elimination of coercive measures in psychiatry? (Prinsen and van Delden, 2009) [41]	Netherlands	Discuss the practice of coercive measures in psychiatry, “addressing the conflict between autonomy and beneficence/non-maleficence, human dignity, patients’ experiences, and the effects of coercive measures” (p. 69).	Discussion paper	“An appeal to respect for autonomy and/or human dignity cannot be a sufficient reason to reject imprisonment; the complete lack of controlled trials on the beneficial effects of coercive measures in different populations argues against the use of coercive measures” (pp. 69–70)
Community Treatment Orders in Psychiatry Confront Principalism: Considerations Reflected in the Light of the Convention on the Rights of Persons with Disabilities (CRPD). (Newton-Howes, 2019) [42]	New Zealand	Discuss whether involuntary outpatient treatment, “as an example of coercive psychiatric practice, opposes the principles of principalism in the modern context” (p. 126).	Discussion paper	Evidence for the clinical efficacy of “involuntary outpatient treatment is marginal, although this has not prevented its increasing application; this issue is receiving closer scrutiny in mental health with the application of the CRPD” (p. 132).
Ethical challenges of inpatient psychiatric wards: a qualitative study of the experiences of Norwegian mental health professionals. (Haugom et al., 2019) [43]	Norway	Examine how clinical staff in psychiatric inpatient wards describe and assess the ethical challenges of incarceration.	Original research Qualitative inquiry	“Ethical challenges seem to be at the heart of the practice of confinement; systematic ethical reflections are a way to process the ethical challenges the team encounters” (p. 1). The findings demonstrate the ethical and burdensome nature of assuming control over the patient, which can lead to psychosocial pressure on the personnel.
Coercive measures in psychiatry: a review of ethical arguments. (Chieze et al., 2021) [44]	Switzerland	Map the ethical elements used in the literature to justify or reject the use of coercive measures that limit freedom of movement, highlighting some important issues.	Review article	Coercive measures decided after a transparent and carefully balanced evaluation process are more likely to be appropriate, understood, and accepted by patients and caregivers.
Mandatory interventions in severe and persistent mental illness: a survey of attitudes among psychiatrists in Switzerland. (Stoll et al., 2021) [45]	Switzerland	Survey attitudes towards palliative care, medical care in death, and involuntary treatment of patients with severe and persistent mental illness.	Original research Cross-sectional survey	Most respondents respect the autonomy of patients with severe and persistent mental illness; however, many found it necessary to perform involuntary interventions.
Coercive care and human rights: a complex juxtaposition—part 1. (Little, 2019) [46]	New Zealand	Explore the clinical implications associated with the United Nations Convention on the Rights of Persons with Disabilities (UN–CRPD) and coercive practice.	Discussion paper	Both human rights and clinical perspectives are needed in interventions with the mentally ill.
Coercion in psychiatric care: can paternalism justify coercion? (Seo et al., 2013) [47]	South Korea	Analyze whether coercive interventions in mental health can be justified by the basic assumptions of paternalists (incompetence, dangerousness, and disability).	Original research Cross-sectional survey	The use of coercive measures to prevent harm to oneself and others must be limited to cases where there is a clear and objective prediction of potential harm and follow the principle of the minimum use of force.
Substance use disorder and compulsory commitment to care: a framework for ethical decision-making in care. (Nicolini et al., 2018) [48]	Belgium	Explore the value of care ethics as a guide for decision-making about involuntary treatment in patients with substance use disorders.	Discussion paper	Decision-making is seen as an “important part of a dynamic care process in which the lived experience, the interpretative dialogue, and the promotion of dignity are central” (p. 1); the patient participates in defining his needs, reducing his perception of coercion, and increasing his motivation for treatment.
Beneficial Coercion in Psychiatric Care: Perceptions of the African Ethical-Cultural System. (Ewuoso, 2018) [49]	South Africa	Propose a new ethical framework for the application of coercion in psychiatric care that respects human dignity.	Discussion paper	Only “a more respectful approach to the application of coercion in psychiatric care can lead to a careful balancing of the conflicting interests of individual rights, individual welfare, and public safety” (p. 91).
Psychiatrists’ motives for practising in-patient compulsory care of patients with borderline personality disorder (Lundahl et al., 2018) [50]	Sweden	Investigate psychiatrists’ motives for involuntary care of patients with Borderline Personality Disorder.	Original research Qualitative inquiry	The study indicates that the practice of involuntary measures in patients with Borderline Personality Disorder differs significantly, depending on the evaluating psychiatrist’s personal judgments and values and not on clinical guidelines or legal guidelines.
Community treatment orders: the ethical balance in community mental health. (Snow and Austin, 2009) [51]	Canada	Identify and discuss some of the most pressing ethical issues in the practice of community involuntary treatment.	Discussion paper	The ethical debate of involuntary community treatment involves balancing individual rights to self-determination with the desire to protect patients and the public from harm.
Differentiate between rights-based and relational ethical approaches. (Trobec et al., 2009) [52]	Slovenia	Identify elements that affect the ethical behavior of nurses and its differentiation in a relational and rights-based ethical approach.	Original research Cross-sectional survey	Nurses with a bachelor’s degree differentiated between the two approaches better than nurses without a degree. Overall, participants emphasized ethics and personal values, but more than half were confused between the two approaches, justifying investment in formal education.
Community treatment orders: exploring the paradox of personalization under compulsion. (Banks et al., 2016) [53]	UK	Explore the experiences of professionals and users of involuntary outpatient treatment and inform about good practices.	Original research Qualitative inquiry	Involuntary outpatient treatment is considered by professionals as necessary and, in some cases, the most useful tool at their disposal to encourage treatment adherence; it was considered a ‘platform’ for accessing social support, while users tended to express a ‘reluctant acceptance’ that it offers greater autonomy compared to involuntary hospitalization; professionals should have more opportunities to share knowledge.
When coercion moves into your home’—a qualitative study of outpatient experiences in Norway. (Riley et al., 2014) [54]	Norway	Develop a narrative study focusing on patients’ perspectives on involuntary outpatient treatment.	Original research Qualitative inquiry	Coercion was experienced as limiting freedom of action through excessive control and little patient influence or participation in their treatment.
Ethical challenges in connection with the use of coercion: a focus group study of health professionals in mental health care. (Hem et al., 2014) [55]	Norway	Analyze the ethical challenges faced by health professionals in their daily clinical work related to the use of coercion.	Original research Qualitative inquiry	A systematic focus on ethical challenges related to coercion is an important step toward improving healthcare in the field of mental health. The difficulty faced by healthcare workers in effectively expressing ethical dilemmas can be attributed to a lack of theoretical resources and a corresponding absence of normative vocabulary that can shed light on these issues in a meaningful manner.
The experiences of detained mental health service users: issues of dignity in care. (Chambers et al., 2014) [56]	UK	Explore, in detail, users’ experiences of detention and coercive interventions.	Original research Qualitative inquiry	“Dignity and respect are important values in recovery, and professionals need time to engage with patients’ narratives and reflect on the ethics of their practice” (p. 1). Data evidence indicates that there was tension over the favorable interactions with personnel, overall care, and/or participation in decision-making related to care planning.
A qualitative study examining the presence and consequences of moral frames in the experiences of patients and mental health professionals of Community Treatment Orders (CTO). (Lawn et al., 2015) [57]	Australia	Understand how the meaning of involuntary outpatient treatment is constructed and experienced from the perspective of patients and professionals directly involved in CTO.	Original research Qualitative inquiry	The experiences are multifaceted, varied, and critically dependent on the empathy and self-reflection of patients and workers; a robust ethical debate, including a reflection on present moral frameworks, is needed in order to overcome the paradox of the moral gray zone between caring and controlling.
Ethical deliberations about involuntary treatment: interviews with Swedish psychiatrists. (Sjöstrand et al., 2015) [58]	Sweden	Explore the ethical reasoning and experiences of Swedish psychiatry about involuntary treatment, addressing the issue of patient autonomy.	Original research Qualitative inquiry	Involuntary treatment was generally seen as an unwanted exception, leaving the law room for individual judgments; cases involving suicidal and psychotic patients were considered paradigmatic of involuntary care; autonomy was sometimes considered a reason for involuntary treatment in order to promote autonomy.
Staff normative attitudes towards coercion: the role of moral doubt and professional context—a cross-sectional research study. (Molewijk et al., 2017) [59]	Norway	Discover “mental health professionals’ normative attitudes towards coercion and how often they experience moral doubts”; how the (lack of) experience of moral doubt is related to the team’s normative attitudes towards the use of coercion; assess “the extent to which individual and professional aspects were associated with the normative attitude and the experience of moral doubt” (p. 2).	Original research Cross-sectional survey	The experience of moral doubt is related to the normative attitude towards coercion; the more professionals are involved in situations of coercion, the more they consider that coercion can be understood as care and security; psychologists were more critical about coercion.
The role of ethics in reducing and improving the quality of coercion in mental health care. (Norvoll et al., 2017) [60]	Norway	Explore health professionals’ descriptions of their ethical challenges and strategies in everyday life to ensure morally justified coercion and best practices.	Original research Qualitative inquiry	Coercion involves individual and institutional ethical aspects; various types of moral deliberation and ethical support can contribute to addressing the challenges of coercion by offering more systematic ways of dealing with moral issues.
Coercive care in mental health—dilemmas in the decision-making process. (Berge et al., 2018) [61]	Norway	Analyze the factors that psychiatric screening clinicians consider important when considering the use of coercive care.	Original research Qualitative inquiry	Psychiatric screening of clinicians in this study highlighted measures that can be used to help involve the patient in the decision-making process. The findings emphasized strategies that may be employed to actively involve the patient in the decision-making process: (a) allocate an adequate amount of time; (b) provide the patient with an opportunity to discuss their situation and articulate their perspectives and any objections; (c) establish a positive connection and partnership with the patient; (d) dedicate time to elucidate the rationale behind actions, proceed at a measured pace, reiterate crucial inquiries, and construct a comprehensive framework of care; (e) attentively listen to and genuinely consider the patient’s input.
The importance of ethical reflection groups in mental health care: a focus group study among health professionals. (Hem et al., 2018) [62]	Norway	Evaluate the importance of participating in systematic ethical reflection groups focused on ethical challenges related to coercion.	Original research Qualitative inquiry	Health professionals in this project “were satisfied with the systematic ethical reflection related to the use of coercion; the ethical reflection groups had positive effects on the coercion” dilemmas addressed by the groups (p. 1).
Patients’ moral opinions about coercion in mental health. (Norvoll and Pedersen, 2018) [63]	Norway	Increase understanding of patients’ opinions and moral considerations regarding coercion.	Original research Qualitative inquiry	The study stresses the importance of institutional factors and alternative voluntary treatment opportunities, as well as the legal and ethical principles of proportionality and intentionality in moral assessments of coercion.
Ethical practice in emergency psychiatry: common dilemmas and virtue-informed navigation. (Hamm, 2021) [64]	USA	Consider approaches that can promote and enhance ethically informed practice in emergencies.	Discussion paper	Ethical practice in these contexts “can be promoted by identifying relevant ethical and legal considerations and guidance and further optimized by overarching virtues that complement legal obligations and ethical principles” (p. 627).
Family Members’ Existential and Moral Dilemmas of Relatives with Coercion in Mental Health. (Norvoll et al., 2018) [65]	Norway	Explore family members’ existential and moral dilemmas regarding coercion and the factors that influence these dilemmas.	Original research Qualitative inquiry	Coercion can reduce but also increase the burden on the family, creating tensions in family relationships, dilemmas, (moral) suffering, and retrospective regrets.
Compulsory hospitalization of patients with mental disorders: state-of-the-art on ethical and legislative aspects in 40 European countries. (Wasserman et al., 2020) [66]	Sweden	Research involuntary admission procedures for patients with mental disorders in 40 countries.	Original research Cross-sectional survey	Legal reasons for involuntary admission should be reformulated to remove patient stigmatization; it is critical to raise awareness of involuntary admission procedures and patient rights; communication about procedures should be widely available; professionals in the field need to be constantly aware of the ethical challenges surrounding involuntary admissions.
Compulsory community treatment in mental health: literature review. (O’Brien et al., 2009) [67]	New Zealand	Review mandatory community treatment, focusing on its historical development and research evidence on its implementation.	Review article	The arguments in favor of autonomy must be pitted against the arguments in favor of the State’s use of its *patriotic* powers. The analysis highlights the ambivalence observed in studies examining the viewpoints of patients and clinicians about community treatment orders.
Mandatory treatment in psychiatry. (Sheehan, 2009) [68]	UK	Review recent studies on the use of compulsive measures in the hospital, in the community, and in special populations.	Review article	With ethical concerns generally challenged by the argument that compulsive measures can lead to beneficial clinical outcomes, the therapeutic alliance has been identified as a significant factor in influencing the impact of compulsory therapy, such as forced medication and seclusion.
Community Treatment Orders: Locating the Social Worker’s Space. (George, 2011) [69]	USA	Analyze Community Treatment Orders (CTO), using examples from professional social work practice.	Discussion paper	CTOs are not a perfect solution to a sticky situation and must be implemented with great regard for the rights of the customer; efforts should be made to keep customers in their community.
Choosing to limit choice: self-binding directives (SBD) in Dutch mental health care. (Berghmans and van der Zanden, 2012) [70]	Netherlands	Discussion of various issues of broad relevance, particularly the legal regulations and jurisdiction of self-binding.	Discussion paper	If coercive care is provided based on the SBD, the experiences of the patient and the psychiatric professional(s) must be discussed, and a possible revision of the SBD and the patient’s historical values can occur.
Coercion in a locked psychiatric ward: Patient and staff perspectives. (Larsen and Terkelsen, 2014) [71]	Norway	Assesses how patients and staff in a Norwegian psychiatric ward experience coercion.	Original research Qualitative inquiry	Professionals who are physically and emotionally close to the patient are more likely to understand the patient as a unique person with individual needs.
A comparison of mental health legislation in five developed countries: a narrative review (Cronin et al., 2017) [72]	Ireland	Describe similarities and differences in mental health legislation across five jurisdictions.	Review article	Broadly similar procedures are used for admitting, detaining, and treating involuntary patients; there are differences regarding the criteria for defining mental disorders, the occurrence of automatic review hearings after involuntary admission, and the role of decision-making supported by health legislation.
Convergence and divergence: An analysis of mechanical constraints. (Jacob et al., 2019) [73]	Canada	Analyze the experience of using mechanical restraints, both from the perspective of the patient and the nursing team responsible for their application.	Original research Qualitative inquiry	Mechanical restraints were considered necessary for the practice and justified within a discourse of risk and safety; however, the emotional reaction of nurses to restraint was similar to that of patients, as nurses experience cognitive dissonance as they get involved in practices that may conflict with how they think or feel; it is important to evince the tensions associated with these interventions and encourage the search for alternatives.
Reducing coercion in mental health. (Sashidharan et al., 2019) [74]	UK	Examine the extent and nature of coercive practices in mental health and consider the ethical and human rights challenges faced by current clinical practices in this area; assess the effectiveness of attempts to reduce coercion and make specific recommendations for making mental health less coercive and more consensual.	Review article	Coercion in its various forms is built into mental health; examples of good practice in this area are limited, and there is almost no evidence regarding the spread or sustainability of individual programs; all forms of coercive practices are inconsistent with human rights-based mental health care.

## Data Availability

This paper is a part of the doctoral thesis of the first author, and all data generated or analysed during this study are included in this article.
